# Posterior reversible encephalopathy syndrome in patient of severe preeclampsia with Hellp syndrome immediate postpartum

**DOI:** 10.11604/pamj.2015.21.60.5546

**Published:** 2015-05-26

**Authors:** Moulay Abdellah Babahabib, Ibrahima Abdillahi, Farid Kassidi, Jaouad Kouach, Driss Moussaoui, Mohammed Dehayni

**Affiliations:** 1 Department of Gynecology-obstetrics, Military Training Hospital Mohamed V, Rabat, Morocco

**Keywords:** Encephalopathy, preeclampsia, Hellp syndrome

## Abstract

Posterior reversible encephalopathy syndrome (PRES) is a rare clinico-neuroradiologic condition, not commonly reported in the literature. PRES is an uncommon complication of severe preeclampsia/eclampsia. We report the management of one patient with postpartum preeclampsia as an association of HELLP syndrome presenting with status-epileptics. Early diagnosis along with timely supportive therapy resulted in the successful management of this challenging case. Recent understanding on the pathophysiology of this uncommon condition is discussed. We highlight the importance to obstetricians, intensive-care physicians and anesthesiologists of recognizing such cases.

## Introduction

Posterior reversible encephalopathy syndrome (PRES) is a well-recognized, clinical and neuro-radiological entity first described in 1996 by Hinchey et al. [1].the first named this syndrome was reversible Posterior leukoencephalopathy syndrome [1, 2]. The PRES is a clinical and radiological entity associating varying degrees, headaches, impaired consciousness, seizures and visual disturbances to neurological and radiological abnormalities of the parietal-occipital white matter [3]. PRES has a unique MRI and CT imaging appearance, which is demonstrated as subcortical and gyral T2-weighted and fluid attenuated inversion recovery (FLAIR), signal hyperintensities that become more diffuse as the extent [2].

## Patient and observation

**Figure 1:**
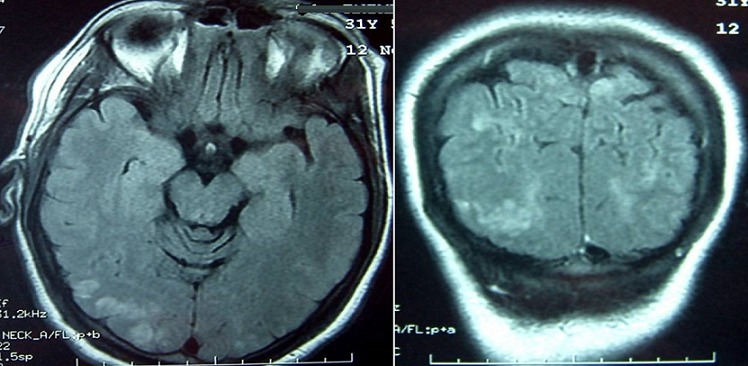
Brain MRI, hypersignal T2 and flair sequences, interesting the cerebral cortex, parietal and occipital sub-corticale

**Figure 2:**
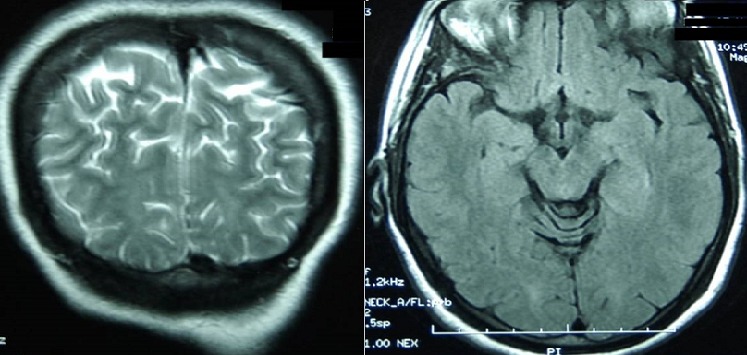
Shows the brain MRI controle did 3 months later with disappearance the cerebral lesions

A woman aged 31 years, G2P2, without any pathological past history, with good prenatal care, with a normal prenatal analysis, blood pressure during the follow-up was normal, admitted for elective caesarian section at 38 weeks of amenorrhea. During realization of the spinal anesthesia the patient presented peak of the hypertensive (180/100 mm Hg) which normalized after a few minutes without treatment. In post-partum, after 12 hours of the caesarian section the lady presented a severe headache with apyrexia then she developed three episodes of generalized tonico-clonic convulsions. The 1^st^ convulsion ceased 1 min after the measures of resuscitation and injection of diazepam IV. The post-critical clinical examination found an afebrile patient and blood pressure at 140/90 mmHg and with Glasgow coma scale at 14 without any neurological deficit, there was no neck rigidity, diuresis was preserved. The examination of urines by urinary strip was positive (+ + +). The 2^nd^ and 3^th^ convulsive crisis stoped spontaneously within few seconds. Laboratory findings of HELLP syndrome include raised liver enzymes (ASAT 525 IU/L and ALAT 214 IU/L), hemolysis (hemoglobinemia in 7 mg/dl) and low platelet count (44 000 platelet/mL). Other investigations included the renal function tests, the inflammatory markers, the thyroid hormones were normal. The viral serology was negative. The patient was transferred to intensive care unit; she was treated immediately by magnesium sulfate associated to calcium channel blocker (nicardipine), antiepileptic (phenobarbital) and preventive dose of low molecular weight heparin. The anemia and thrombopenia corrected by transfusion of packed red blood cells and platelet respectively. The magnetic resonance imaging (MRI), realized one hour after the first episode of convulsion showed zones in hyposignal T1, hypersignal T2 and flair sequences, interesting the cerebral cortex, parietal and occipital sub-cortical and the white matter. Intracranial venous sinuses were permeable. The diagnosis of the PRES syndrome secondary to the severe preeclampsia immediate post-partum was retained (Figure 1). The electroencephalogram (EEG) realized in the second day did not showed anomaly. The evolution was marked by the normalization of blood pressure, the normalization of the neurological state and of the biological analysis. The patient discharged in good condition on day 5, with beta-blocker and sodium valproate maintained during three months. THE MRI of the controle made three months later showed complete resolution of cerebral edema Figure 2.

## Discussion

The pathophysiology of PRES remains unclear and is a disease entity of multifactorial etiologies. Although the majority of patients present severe hypertension, others only show mild hypertension or normal blood pressure. Hypertensive encephalopathy and eclampsia which are both common causes of PRES are thought to have similar pathophysiology, clinical features and imaging finding [4, 5]. On the pathologic level, we suggest one dysfunction of the blood brain barrier by passing self-capacity of cerebral pressure perfusion or endothelial cytotoxicity, responsible for the alteration of the blood brain barrier (BBB). These two mechanisms are held responsible for a vasogenic edema in the white matter, involving the clinical and radiological manifestations of PRES [3, 6, 7]. In the case of perpartum, there are other additional mechanisms, especially capillary hyperpermeability of pregnancy, blood pressure and the role of substances modulating the vascular tone such as: hypersensitiveness to endogenous vasopressor agents, the reduction of prostaglandins and cytotoxic factor of placental origin, responsible for endothelial cell dysfunction [3]. On the topographic plan, the posterior circulation is more susceptible to infarction compared to the anterior circulation; some suggest that this is due to the reduced amount of sympathetic innervation in the posterior circulation over the anterior circulation [5].

PRES a recently described clinical-neuroradiological term that is associated with several medical conditions besides preeclampsia/eclampsia and hypertension e.g. renal failure, post-transplantation (Allogeneic bone marrow transplantation; solid organ transplantation), immunosuppressive therapy (Cyclosporine; Tacrolimus) autoimmune diseases (SLE; systemic sclerosis; Wegener's granulomatosis; poly arteritis nodosa), post-cancer chemotherapy and has recently shown to be associated with infection, sepsis, and shock [6, 7]. There are a range of neurological presentations that often involve generalized seizures, sometimes complicated by status epilepticus, in combination with headaches, confusion, and nausea and vomiting. There may be a focal neurological deficit, such as cortical blindness, cerebellar syndrome, or hemiparesis. These presentations may lead to coma [8]. Our patient presented brief of peak hypertension followed severe headache and three successive episodes of generalized tonico-clonic convulsions. The differential diagnosis for seizures in the post-partum period includes eclampsia, subarachnoid haemorrhage, intracerebral haemorrhage, thrombotic phenomena, intracranial neoplasm, head trauma, idiopathic epilepsy, infection (meningo-encephalitis), amniotic fluid embolism, postpartum angiopathy [1]. The radiologic findings may be seen in an unenhanced Computed Tomography scan (CT scan) but MRI is a key element of diagnosis. It helps to rule out other possibilities of diagnosis: myocardial venous and arterial cerebral thrombophlebitis as well as brain encephalitis. Indeed, it expresses abnormalities of parietal, occipital and frontal cerebral cortex and even in cortical signal, hypo intensive in T1, hyper intensive in T2 and FLAIR (fluid-attenuated inversion recovery). The fast regression of the lesions showed a vasogenic edematous character of the lesions confirming the diagnosis of PRES [3, 7]. The MRI findings in our patient shows zones in hypo signal T1, hyper signal T2 and flair sequences, interesting the cerebral cortex, parietal and occipital sub-cortical and the white matter. Intracranial venous sinus was permeable.

The mainstay of treatment of PRES is recognition and removal of the precipitating factors e.g.: control of blood pressure. Some physicians use antiepileptic drugs or magnesium sulfate to avoid the progress of seizures [6, 9]. Our patient was administrated immediately magnesium sulfate and antiepileptic (Phenobarbital) with good improvement, and was discharged with an oral antihypertensive and antiepileptic. The clinical outcome is variable but is mostly favorable with prompt treatment of the underlying cause and immediate action to identify potential triggering drugs, controlling hypertension, and treating etiology of PRES can lead to complete reversal of radiological and neurological findings. However, in few patients, PRES progresses to ischemia, infarction, or death [1, 6].

## Conclusion

PRES of postpartum is still largely unknown, although it is relatively common. Its pathophysiology remains controversial, MRI is crucial for its diagnosis, and itis usually reversible. The list of conditions known to be associated with PRES is increasing steadily and studies are needed to evaluate the characteristics of PRES associated with postpartum.
